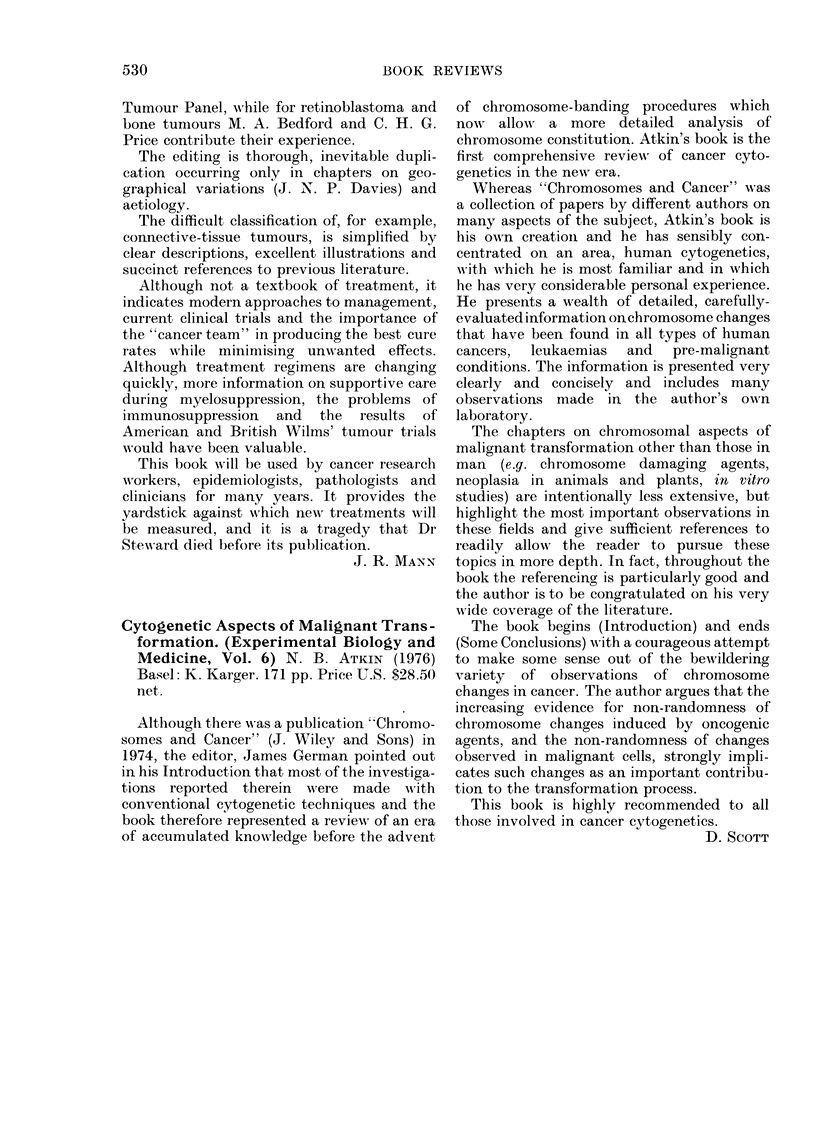# Cytogenetic Aspects of Malignant Transformation. (Experimental Biology and Medicine, Vol. 6)

**Published:** 1977-10

**Authors:** D. Scott


**Cytogenetic Aspects of Malignant Transformation. (Experimental Biology and Medicine, Vol. 6)** N. B. Atkin (1976) Basel: K. Karger. 171 pp. Price U.S. $28.50 net.

Although there was a publication "Chromosomes and Cancer" (J. Wiley and Sons) in 1974, the editor, James German pointed out in his Introduction that most of the investigations reported therein were made with conventional cytogenetic techniques and the book therefore represented a review of an era of accumulated knowledge before the advent of chromosome-banding procedures which now allow a more detailed analysis of chromosome constitution. Atkin's book is the first comprehensive review of cancer cytogenetics in the new era.

Whereas "Chromosomes and Cancer" was a collection of papers by different authors on many aspects of the subject, Atkin's book is his own creation and he has sensibly concentrated on an area, human cytogenetics, with which he is most familiar and in which he has very considerable personal experience. He presents a wealth of detailed, carefully-evaluated information on chromosome changes that have been found in all types of human cancers, leukaemias and pre-malignant conditions. The information is presented very clearly and concisely and includes many observations made in the author's own laboratory.

The chapters on chromosomal aspects of malignant transformation other than those in man (*e.g.* chromosome damaging agents, neoplasia in animals and plants, *in vitro* studies) are intentionally less extensive, but highlight the most important observations in these fields and give sufficient references to readily allow the reader to pursue these topics in more depth. In fact, throughout the book the referencing is particularly good and the author is to be congratulated on his very wide coverage of the literature.

The book begins (Introduction) and ends (Some Conclusions) with a courageous attempt to make some sense out of the bewildering variety of observations of chromosome changes in cancer. The author argues that the increasing evidence for non-randomness of chromosome changes induced by oncogenic agents, and the non-randomness of changes observed in malignant cells, strongly implicates such changes as an important contribution to the transformation process.

This book is highly recommended to all those involved in cancer cytogenetics.

D. Scott